# Using electric current to surpass the microstructure breakup limit

**DOI:** 10.1038/srep41451

**Published:** 2017-01-25

**Authors:** Rongshan Qin

**Affiliations:** 1 Professor in Advanced Materials Engineering, School of Engineering & Innovation, The Open University, Walton Hall, Milton Keynes MK7 6AA, United Kingdom

## Abstract

The elongated droplets and grains can break up into smaller ones. This process is driven by the interfacial free energy minimization, which gives rise to a breakup limit. We demonstrated in this work that the breakup limit can be overpassed drastically by using electric current to interfere. Electric current free energy is dependent on the microstructure configuration. The breakup causes the electric current free energy to reduce in some cases. This compensates the increment of interfacial free energy during breaking up and enables the processing to achieve finer microstructure. With engineering practical electric current parameters, our calculation revealed a significant increment of the obtainable number of particles, showing electric current a powerful microstructure refinement technology. The calculation is validated by our experiments on the breakup of Fe_3_C-plates in Fe matrix. Furthermore, there is a parameter range that electric current can drive spherical particles to split into smaller ones.

Fabrication of nanoscale grains^1^, droplets^2^,^3^ and bubbles^4^ has attracted significant attentions in recent decades. Microstructure refinement toward the generation of nanostructured materials is an attractive mean^5^. In the refinement processing, microstructural elements (grains, droplets, bubbles, etc.) are stretched into long thin plates by severe mechanical deformation followed by breakup into smaller pieces and subsequent spheroidization. Grains in alloys are stretched by rolling, drawing and compression into thin plates^6^, followed by annealing to allow the stretched microstructure to breakup and spheroidized into finer grains^7^. Droplets and bubbles are stretched by shearing, spraying and splashing into thin film^8^, followed by breakup and spheroidization into finer soft particles^9^. The governing principle behind the breakup process is the irreversible thermodynamics, i.e. the minimization of system free energy. Phase transition is not involved in the breakup process when the volume fraction of each phase remains. The total interfacial free energy (*g*_int_ = *σ* · *s*, where *σ* is the interfacial tension and *s* is the total interfacial area) is dominating in the conventional breakup processing. Some materials, e.g. surfactant and micelle^10^, have negative interfacial tension (*σ* < 0). In those materials a spherical droplet may turn itself into cylindrical or split into smaller ones in order to increase the total interfacial area and hence to reduce the total system interfacial energy. However, most other materials (e.g. metals) are with positive interfacial tension (*σ* > 0). The breakup is limited by the interfacial area minimization (*δs* ≤ 0). The breakup limit restrains the microstructure refinement by stretch and breakup processing mean.

Our aim was to introduce an extra free energy term into the system so that the microstructure breakup limit, *δs* ≤ 0, can be lifted up to a new level. Electric current free energy (*g*_*elec*_) is different from the chemical free energy in a way being dependent on the microstructure configuration. The breakup causes the change of electric current free energy (*δg*_*elec*_). The irreversible thermodynamics gives the new breakup limit as *δs* + *δg*_*elec*_ < 0. When *δg*_*elec*_ < 0, it allows *δs* > 0 as far as *δs* < −*δg*_*elec*_. The stretched thin plates or thin films are able to break up into more number of finer pieces with each piece has smaller diameter after spheroidization. This pushes the refinement processing toward the fabrication of much finer microstructure.

In the present work, a breakup of high electrical resistivity phase in a low electrical resistivity matrix is considered. The real materials parameters are implemented in the calculation for a purpose of comparison with experimental validation. The work focuses on thermodynamics. The results are generic for the breakup of any grains, droplets and bubbles submerged in a higher conductive matrix.

## Results

**Figure 1:**
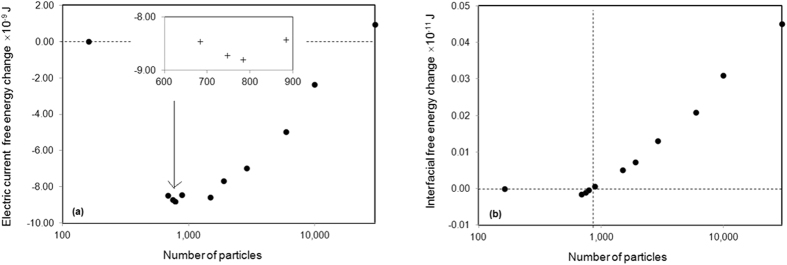
Change of free energy when 163 ellipsoid grains break up into various numbers of spherical particles. (**a**) The change of electric current free energy, where the inserted figure shows the reduction of free energy when the particle numbers are increasing from 682 to 782. (**b**) The change of interfacial free energy, where the vertical dashed line points out the breakup limit.

**Figure 2:**
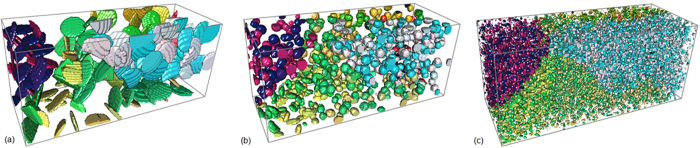
Thin plates break up into different numbers of particles, where the volume fraction of the grains remains at 12.21 vol.%. (**a**) 163 ellipsoid grains. (**b**) 745 spherical particles. (**c**) 29,882 fine particles.

Figure 1 shows the calculated change of electric current free energy and the change of interfacial free energy during the breakup of 163 ellipsoid grains into various numbers (from 682 to 29,882) of spheroid particles in a matrix. All the grains added together to occupy 12.21 vol.% (experimental value) of the space. The geometry, orientation and distribution of the ellipsoid grains before breakup and the spherical particles after broken up are demonstrated in Fig. 2, where colour represents the crystallographic orientation^11^. The matrix is assumed to be ferritic iron which has electrical conductivity 9.17 × 10^6^ S · m^−1^ and magnetic permeability 300*μ*_*0*_ at ambient conditions, where *μ*_0_ = 1.26 × 10^−6^*N* · *A*^−2^ is the vacuum permeability^12^,^13^,^14^,^15^. The ellipsoid grains are assumed to be Fe_3_C cementite crystals which has electrical conductivity 1.22 × 10^6^ S · m^−1^ and magnetic permeability 30*μ*_*0*_^12^,^13^,^14^,^15^. The electrical resistivity of interface is assumed to have the average value between that of the adjacent phases. This is equivalent to a sharp interface approximation so that the resistance is equivalent to that of the serial connection between two resistors. The Curie temperature for ferritic iron and Fe_3_C cementite is 1043 K and 480 K, respectively. Without losing generality, the input parameters were selected in such a way that experimental validation can be performed easily in our laboratory. The results, however, are able to indicate the generic breakup of the higher electrical resistivity plates inside a lower electrical resistivity matrix. Numerical calculations were performed on 200 × 90 × 80 cubic grid with a lattice distance 10 nm. An electrical potential of 20 volts were applied to the sample along the longest axis. The interfacial tension between ferrite iron and Fe_3_C is 2.8 J · m^−2^ according to literature^16^. Figure 1(a) shows a reduction of electric current free energy when 163 ellipsoid grains break up and form <22,000 spherical particles. Figure 1(b) shows that the breakup limit is less than 882 spherical particles if no electric current is implemented. In this case, the breakup limit in terms of particle number is increased from 882 to 22,000 (roughly 25 times). More interestingly, Fig. 1(a) shows that when the number of spherical particles increases from 682 to 745 and then to 782 the electric current free energy is in a trend of reduction. This means that some particles can split into smaller ones in the parameter range. This is not at all possible in the convention breakup process when *σ* > 0, where the interfacial free energy increases along the particle numbers monotonically, as that showing in Fig. 1(b).

Figure 1 shows that the change of electric current free energy amplitude is around 4 orders of magnitude higher than that of the interfacial free energy in the considered parameters. The calculation reveals that the desirable electric current density to enable the breakup of 162 ellipsoid particles to 22,000 spherical is around 10^11^*A* · *m*^−2^. In many electronic devises, e.g. the reading and writing system in computer hard disk^17^, the peak electric current density is between 10^9^*A* · *m*^−2^ and 10^14^*A* · *m*^−2^. Structural instability may happen from the thermodynamic point of view if those devises are with the similar microstructure. When the system is scaled up from Δ*x* = 10 nm to Δ*x* = 10 μm while the rest parameters remain unchanged, the electric current free energy is magnified by a factor of Δ*x*^3^ but the interfacial free energy is magnified by a factor of Δ*x*^2^. The desirable electric current free energy is reduced by a factor of 10^−4^, which indicates a change of critical electric current density by a factor of 10^−2^ because the free energy is proportional to the current density square^18^,^19^. The critical current density to break the breakup limit is 10^9^*A* · *m*^−2^.

**Figure 3:**
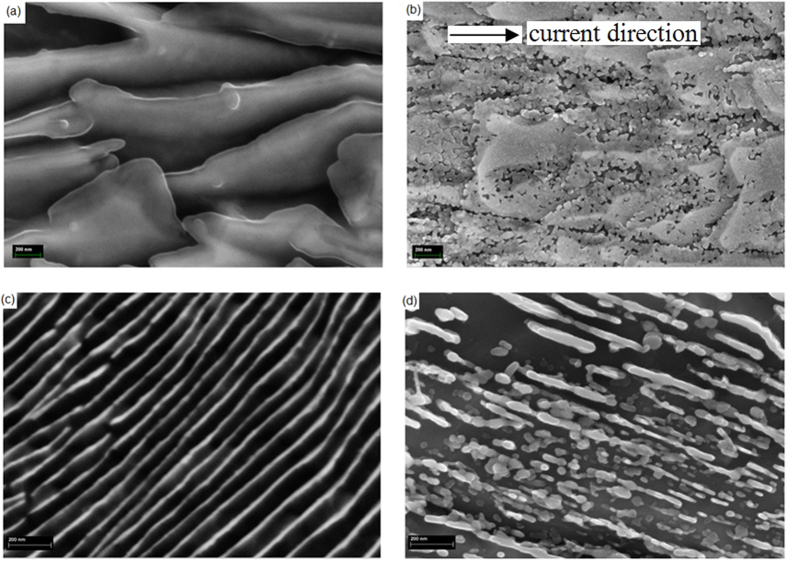
Scanning electron microscope images show the electric-current-driven Fe_3_C stretched thin plates to break up into fine grains. (**a**) Before electric current treatment in longitude view. (**b**) After treatment in longitude view. (**c**) Before treatment in transverse view. (**d**) After treatment in transverse view.

Figure 3 presents the scanning electron microscope images for the experimental observation of passing electric current of around *9* × *10*^*9*^*A* · *m*^−*2*^ to a cold drawn pearlitic steel rod of 15 cm long and 2.84 mm diameter at ambient temperature and pressure. The strained cementite plates break up into nanoscale particles without change the integrity of the materials. Figure 3(a) and (b) are the longitude view of the microstructure before and after the passing of an electric current pulse, respectively, while Fig. 3(c) and (d) are the transverse view of the microstructure before and after the electric current treatment, respectively. The microstructure is extremely fine and has not been reported obtainable using other conventional breakup refinement methods. The temperature rising due to passing electric current is less than 100 °C, due to very short pulse duration rather than direct current.

## Discussion

The electric current free energy is represented as^18^


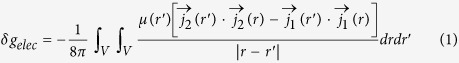


Where *r* is a spatial position. 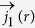 and 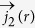 are electric current density at *r* before and after the microstructural breakup, respectively. *μ* is the magnetic permeability. Equation (1) is a modification of its conventional format to suitable for multiphase materials with various magnetic permeability^19^. The equation shows that the change of electric current free energy in the microstructural breakup is due to the associated electric current density redistribution. The chemical free energy has nothing to do with the phase reconfiguration and hence unable to contribute to the microstructure breakup. The distribution of electric current density is calculated using Kirchhoff’s circuit laws. Numerical calculation of electric current distribution and corresponding electric field free energy at steady state gives the results presented in Fig. 1.

**Figure 4:**
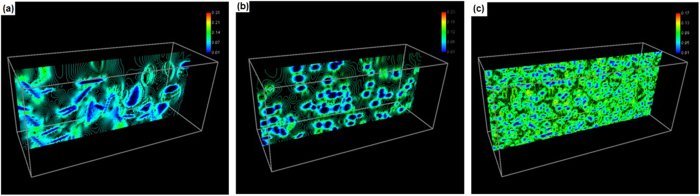
Contour of electric current density in a section of material. (**a**) The microstructure contains 163 ellipsoid grains. (**b**) 745 spherical particles. (**c**) 29,882 fine particles.

**Figure 5:**
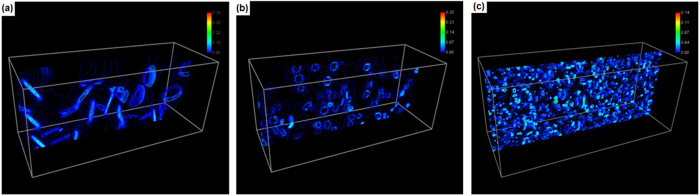
Temperature rising due to passing electric current in a dimension of 
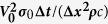
. (**a**) The microstructure contains 163 ellipsoid grains. (**b**) with 745 spherical particles. (**c**) with 29,882 fine particles.

Figure 4 shows the distribution contour of electric current density in a section of material when the microstructure contains 163 ellipsoid grains, 745 spherical particles and 28,882 fine particles, respectively. The scale is in a dimension of *j*_0_ = *V*_0_ · *σ*_0_/Δ*x*, where *σ*_0_ = 1.0 × 10^7^ Sm^−1^ and *V*_0_ = 1 volt. When the ellipsoid particles are broken to form spherical particles, the distribution of local electrical resistivity is changed. The distribution of electric current density is hence altered. It can be seen from Fig. 4 that the electric current density inside Fe_3_C is smaller than that of outside due to its high electric resistivity. Figure 5 is the corresponding temperature rising contour due to passing electric current. The scale is in a dimension of 
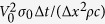
, where Δ*t* is the current load duration, ρ is the mass density of grain and *c* is the specific heat of the suspension phase. It can be seen that the temperature rising inside the high electrical resistivity grain is higher than that of the outside. The higher temperature promotes the carbon diffusion, which accelerate the microstructure transformation such as breakup and spheroidization.

The electric-current-induced surpass of conventional breakup limit is applicable for the breakup of higher electrical resistivity phases in a lower electrical conductivity matrix. This is also suitable for gas bubble and liquid droplets submerged in a high conductive fluid such as plasma, ionic solution or other materials. Electric current helps to surpass the conventional breakup limit and enables to fabricate much finer microstructures.

It is reported in literature that electric field promotes the change of the spherical particles into ellipsoidal ones^20^,^21^. Electric field deforms the droplets^21^ and in some cases reduces interfacial energy^22^. The electric field-induced interface wetability helps to obtain high aspect ratio ellipsoid droplets^23^. For the droplets carrying static charges, e.g. polymer droplets, the electric static force promotes the droplets breakup^24^. The mechanism revealed in the present work provided extra effect to the current-induced breakup processing reported in literature.

It is also known that electric current helps to generate refined microstructure during liquid-liquid (e.g. spinodal decomposition)^25^, liquid-solid^26^ and solid-solid phase transition^27^,^28^. Electric current free energy plays an important role in those phenomena. The reported effect in this work is an additional microstructure refinement mechanism on top of its effects during phase transition. Using of electric-current to manipulation materials microstructure has been proved in some cases^29^,^30^. This will find more applications.

## Methods

### Microstructure construction

The microstructure construction follows phase field scheme^31^,^32^. The initial space is defined as liquid. This is followed by nucleation of 8 face-centered-cubic (fcc) austenite crystals at random position and each with random crystal orientation. The austenite crystals grow with a constant speed until filling all the grids. Fe_3_C crystals and body-centered-cubic (bcc) ferrite crystals are subsequently nucleated and grown from the austenite crystals without penetration of fcc austenite grain boundary. The number of nuclei and growth rate (isotropic or anisotropic growth) of Fe_3_C crystals are input parameters. The orientation between Fe_3_C and bcc ferrite obeys Pitsch-Petch relationship of 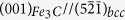, 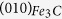 2°–3° from 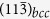 and 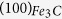 2°–3° from 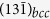
11 The shape of Fe_3_C phase is obtained as either ellipsoid thin plates with an aspect ratio between shortest and longest axis of 0.1 or spherical particles. The volume fraction of Fe_3_C in the material is defined as 12.21% exactly. The particles are not guaranteed to be in the same size due to the compliment of the phase volume fraction but the maximum difference is 1 grid only.

### Electric current free energy calculation

According to Ohm’s law, the local electric current density is determined by the local electrical conductivity *σ*(*r*) and the local electric field 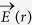 via 
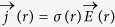
, where *r* is a spatial position in material. The electric field is determined by the gradient of the local electric potential *φ*(*r*) by 
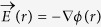
. Gauss’s law represents the relationship between the local electric field and the local electric charge density ρ(r) as 
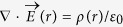
, where ε_0_ the permeability of free space. For the materials considered in the present work, nowhere has net charge. This enables Gauss’s law to reduce to Poission’s equation, as expresses in followings


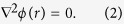


The distribution of the electric potential can be obtained by solving Eq. (2) with adequate boundary conditions. Eq. (2) can also be represented as 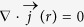. This is equivalent to the statement of Kirchhoff’s current law and can be used as the governing equation for calculating the distribution of electric potential and the distribution of electric current density using relaxation method.

### Experiments

Commercial pearlitic steel wires of chemical composition Fe–0.8C–0.2Si–0.5Mn (wt.%) were received and then cold-drawn to a true strain of 1.61 and cut into 15 cm length samples^33^. An electric current pulse with peak current density 9 × 10^9^ A/m^2^ was implemented to the sample at ambient temperature. The pulse was generated by a capacitor discharging. The wave-shape of the pulse is recorded by an oscilloscope. It is in a damped shape oscillations with initial period around 20 μs and total duration of 110 μs. The peak current was measured based on a calculation using discharging voltage and total resistance in the circuit. Electric current flows along the longitudinal direction of the sample wire from anode to cathode. After the treatment, the sample was cut along the longitudinal and cross-sections, polished and etched in natal for metallographic analysis and examined by Carl Zeiss made Ultra-55 scanning electron microscope (SEM).

### Software

The three-dimensional visual analysis of the computational data was generated using an in-house developed MatVisual software.

## Additional Information

**How to cite this article**: Qin, R. Using electric current to surpass the microstructure breakup limit. *Sci. Rep.* **7**, 41451; doi: 10.1038/srep41451 (2017).

**Publisher's note:** Springer Nature remains neutral with regard to jurisdictional claims in published maps and institutional affiliations.
